# Supplementary material for: RISE: Two‐Stage Rank‐Based Identification of High‐Dimensional Surrogate Markers Applied to Vaccinology
Source: Stat Med. 2025 Sep 5;44(20-22):e70241. doi: 10.1002/sim.70241 (PMC12412727; doi:10.1002/sim.70241)
Supplement: Supplementary file 1 — Data S1: Supporting information may be found in the online version of the article at the publisher's website. R markdown files to fully reproduce all results from this article are available on the GitHub repository github.com/arthurhughes27/RISE‐project. Functions and documentation to apply RISE are available in the R package SurrogateRank, available on the CRAN. [file SIM-44-0-s001.pdf]

Supporting Information for "RISE: Two-Stage Rank-Based Identification of High-Dimensional Surrogate Markers Applied to Vaccinology"

Arthur Hughes[1,2], Layla Parast[3], Rodolphe Thiébaut[1,2,4], and Boris P. Hejblum[1,2]

[1]INSERM Bordeaux Population Health Research Center, INRIA SISTM, University of Bordeaux, F-33000 Bordeaux, France
[2]Vaccine Research Institute, F-94000 Créteil, France
[3]Department of Statistics and Data Science, University of Texas at Austin, Austin, TX 78712, United States
[4]Centre Hospitalier Universitaire de Bordeaux, Service d'Information Médicale, INSERM, Bordeaux F- 33000, France

1 Variance derivations for the paired-sample extension

In this setting, observed data consist of $i = 1, ..., n$ i.i.d observations of primary response $\boldsymbol{Y_i} = (Y_i^1, Y_i^0)^T$ and surrogate candidate $\boldsymbol{S_i} = (S_i^1, S_i^0)^T$. As before, define treatment effects on Y and S respectively as

$$U_Y = \mathbb{P}(Y^1 > Y^0) + \frac{1}{2}\mathbb{P}(Y^1 = Y^0)$$

$$U_S = \mathbb{P}(S^1 > S^0) + \frac{1}{2}\mathbb{P}(S^1 = S^0),$$

Since data are paired, we estimate these by the proportion of individuals for which the treated observation is greater than its control counterpart, i.e.

$$\widehat{U}_Y = n^{-1}\sum_{i=1}^{n} G(Y_i^1, Y_i^0)$$

$$\widehat{U}_S = n^{-1}\sum_{i=1}^{n} G(S_i^1, S_i^0)$$

where

$$G(A, B) = \begin{cases} 1, & \text{if} \quad A > B \\ \frac{1}{2}, & \text{if} \quad A = B \\ 0, & \text{if} \quad B < A \end{cases}$$

We first derive the variance of the estimated U-statistics $\widehat{U}_Y$ (and by extension $\widehat{U}_S$) under the null hypothesis of no treatment effect.

First, note that we only have three possible events : $Y^1 > Y^0$, $Y^0 > Y^1$, $Y^0 = Y^1$. Under the null hypothesis, $P(Y^1 > Y^0) = P(Y^1 < Y^0)$. Let $P(Y^0 = Y^1) = \pi$. Now, since the sum of the probability of all three events must be 1, we have

$$\pi = 1 - 2P(Y^1 > Y^0)$$

$$\implies P(Y^1 > Y^0) = \frac{1 - \pi}{2}$$

Now,

$$\begin{aligned} E(G(Y^1, Y^0)) &= 1 \cdot P(Y^1 > Y^0) + \frac{1}{2} \cdot P(Y^1 = Y^0) + 0 \cdot P(Y^1 < Y^0) \\ &= \frac{1-\pi}{2} + \frac{\pi}{2} \\ &= \frac{1}{2} \end{aligned}$$

To derive the second moment, notice that

$$G(A,B)^2 = \begin{cases} 1, & \text{if} \quad A > B \\ \frac{1}{4}, & \text{if} \quad A = B \\ 0, & \text{if} \quad B < A \end{cases}$$

Then,

$$\begin{aligned} E(G(Y^1, Y^0)^2) &= 1 \cdot P(Y^1 > Y^0) + \frac{1}{4} \cdot P(Y^1 = Y^0) + 0 \cdot P(Y^1 < Y^0) \\ &= \frac{1-\pi}{2} + \frac{\pi}{4} \\ &= \frac{2-\pi}{4} \end{aligned}$$

So, the variance is

$$\begin{aligned} Var(G(Y^1, Y^0)) &= E(G(Y^1, Y^0)^2) - E(G(Y^1, Y^0))^2 \\ &= \frac{2-\pi}{4} - \frac{1}{4} \\ &= \frac{1-\pi}{4} \end{aligned}$$

Then, since individuals are independent, we have

$$\begin{aligned} Var(U_Y) &= Var(\frac{1}{n}\sum_{i=1}^{n} G(Y_i^1, Y_i^0)) \\ &= \frac{n}{n^2} Var(G(Y^1, Y^0)) \\ &= \frac{1-\pi}{4n} \end{aligned}$$

In the case of a truly continuous response, we have $\pi = 0$ and $Var(U_Y) = \frac{1}{4n}$. Otherwise, in the case of ordinal responses we can estimate

$$\widehat{\pi} = \frac{1}{n}\sum_{i=1}^{n} \mathbb{1}(Y_i^1 = Y_i^0)$$

The estimated null variance of the U-statistics in used in order to adaptively choose the non-inferiority threshold ϵ as follows : if the estimated treatment effect is $\widehat{U}_Y$, the significance level α and the desired power to detect a treatment effect based upon the candidate surrogate S is $(1-\beta)$, one may select ϵ as:

$$\epsilon = \max\left\{0, \widehat{u}_Y - u^*_{\alpha,\beta}\right\}, \tag{1}$$

where

$$u^*_{\alpha,\beta} = \frac{1}{2} - \sqrt{\frac{1-\widehat{\pi}}{4n}}\left[\Phi^{-1}(\beta) - \Phi^{-1}(1-\alpha)\right].$$

Now, we use similar arguments to derive the variance of $\widehat{\delta} = \widehat{U_Y} - \widehat{U_S}$.

$$
\begin{aligned}
\widehat{\delta} &= \widehat{U_Y} - \widehat{U_S} \\
&= \frac{1}{n}\sum_{i=1}^{n} G(Y_i^1, Y_i^0) - \frac{1}{n}\sum_{i=1}^{n} G(S_i^1, S_i^0) \\
&= \frac{1}{n}\sum_{i=1}^{n}[G(Y_i^1, Y_i^0) - G(S_i^1, S_i^0)] \\
&= \frac{1}{n}\sum_{i=1}^{n} d_i
\end{aligned}
$$

where $d_i = G(Y_i^1, Y_i^0) - G(S_i^1, S_i^0)$. Then, since individuals are independent,

$$
\begin{aligned}
Var(\widehat{\delta}) &= Var(\frac{1}{n}\sum_{i=1}^{n} d_i) \\
&= \frac{1}{n^2}\sum_{i=1}^{n} Var(d_i) \\
&= \frac{n\sigma_d^2}{n^2} \\
&= \frac{\sigma_d^2}{n}
\end{aligned}
$$

such that $\widehat{\delta} \sim \mathcal{N}(\delta, \frac{\sigma_d^2}{n})$. In practice, we can estimate σ_d with its sample estimator

$$
\widehat{\sigma_d}^2 = \frac{1}{n-1}\sum_{i=1}^{n}(d_i - \bar{d})^2
$$

where

$$
\bar{d} = \frac{1}{n}\sum_{i=1}^{n} d_i
$$

such that $\widehat{Var(\delta)} = \frac{\widehat{\sigma_d}^2}{n}$.

2 Supporting figures and tables

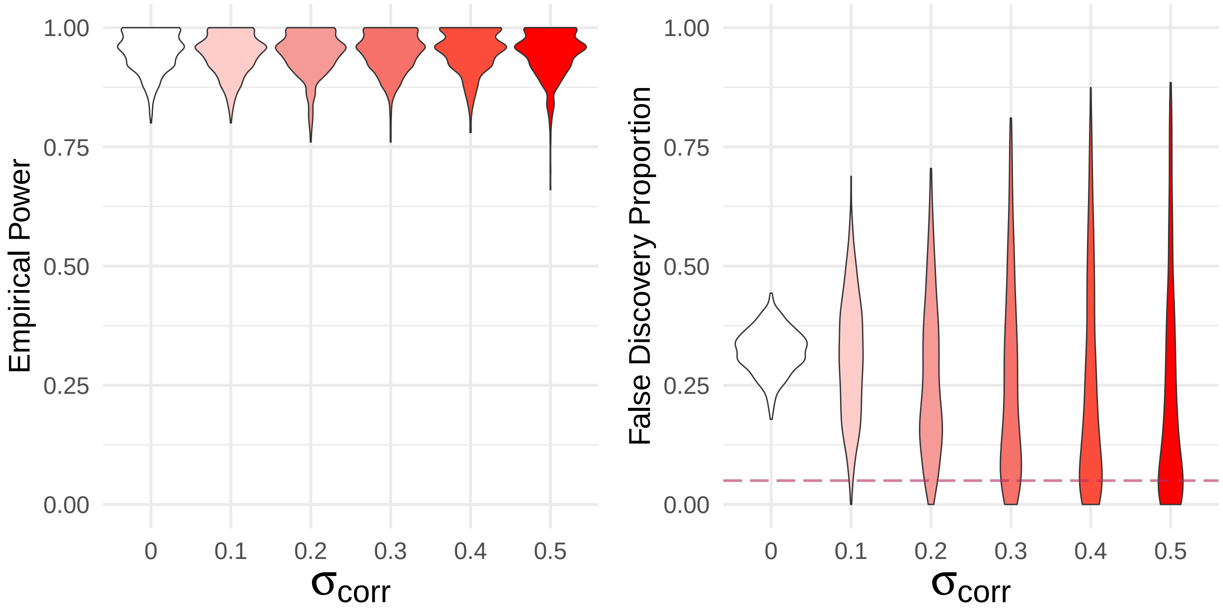

Supporting Information Figure 1: Data generation process 1, scenario 2: violin plots of empirical power (left) and false discovery proportion (right) prior to multiple testing corrections for a fixed sample size $n = 50$ and average surrogate strength $\bar{U_S} = 0.9$ for different values of inter-predictor correlation. The nominal significance level $\alpha = 0.05$ is plotted as a dashed purple line on the FDR plot.

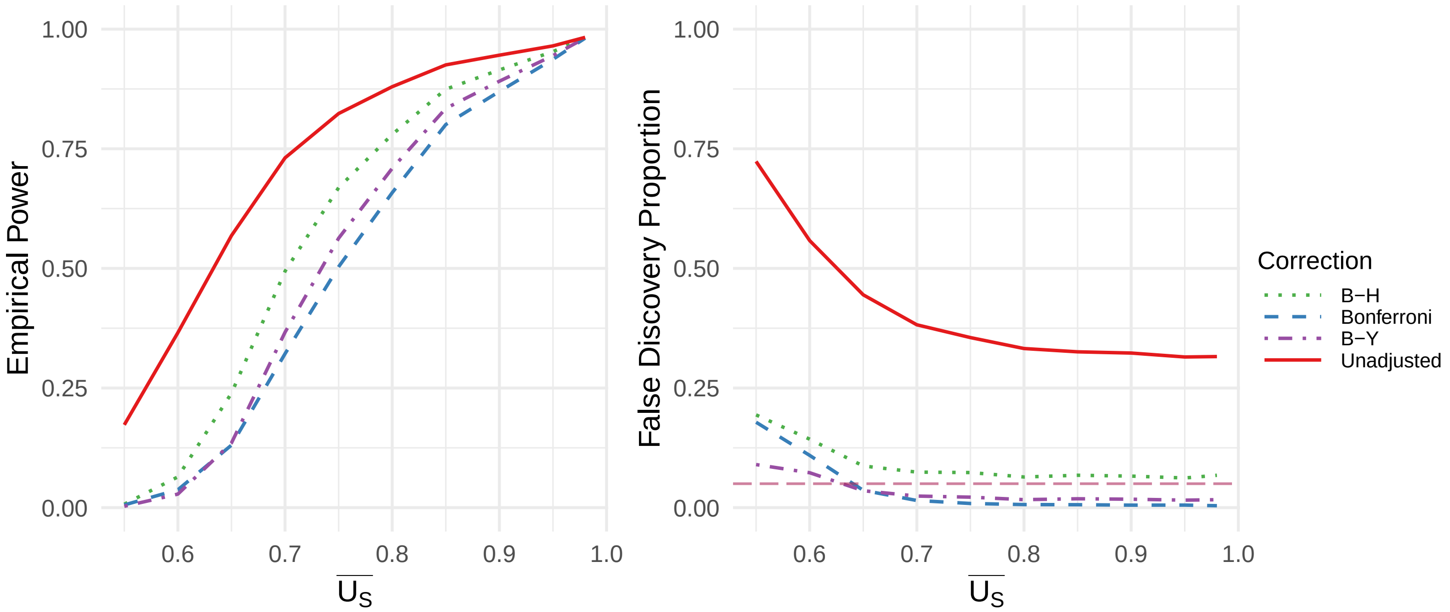

Supporting Information Figure 2: Data generation process 1, scenario 2: Empirical power (left) and false discovery proportion (right) prior to multiple testing corrections as a function of average surrogate strength for different multiple testing corrections (Benjamini-Hochberg, Bonferroni, Benjamini-Yekutieli, Unadjusted) for a fixed sample size $n = 50$. The nominal significance level $\alpha = 0.05$ is plotted as a dashed purple line on the FDR plot.

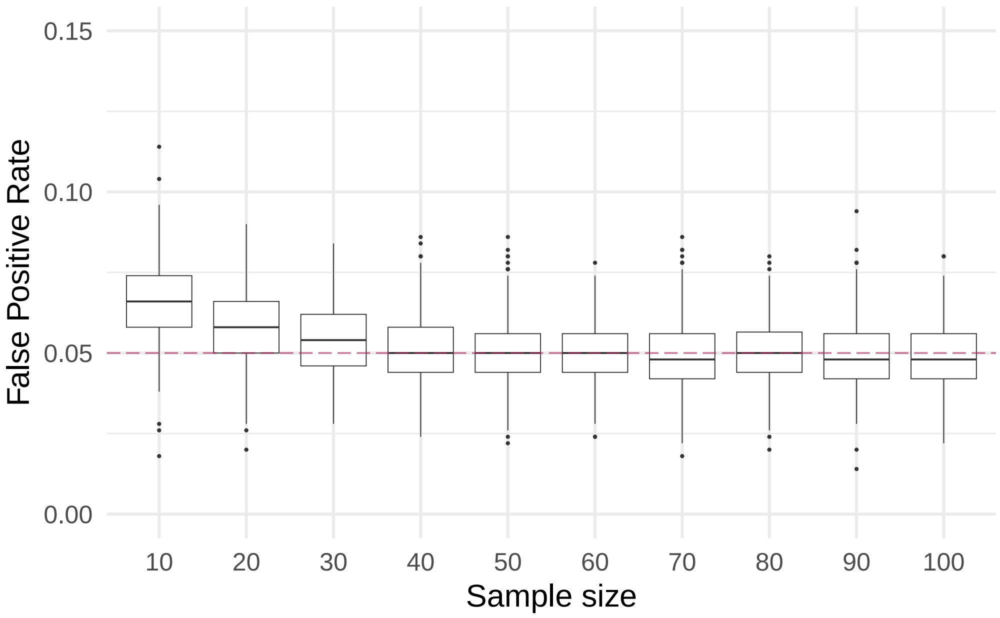

Supporting Information Figure 3: Data generation process 2, scenario 1: boxplots of observed false positive rates against different sample sizes in the uncorrelated setting. The nominal significance level $\alpha = 0.05$ is plotted as a dashed purple line.

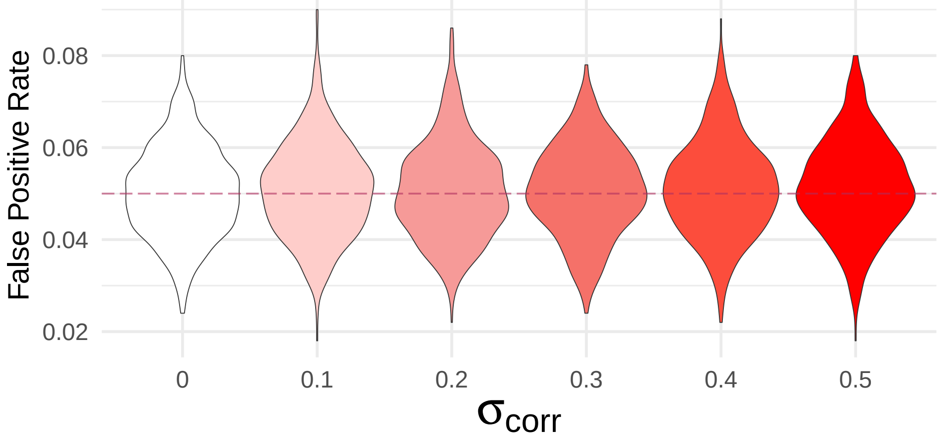

Supporting Information Figure 4: Data generation process 2, scenario 1: violin plots of observed false positive rates against different levels of correlation prior to multiple testing corrections across 500 simulations for a fixed sample size of $n = 50$. The nominal significance level $\alpha = 0.05$ is plotted as a dashed purple line.

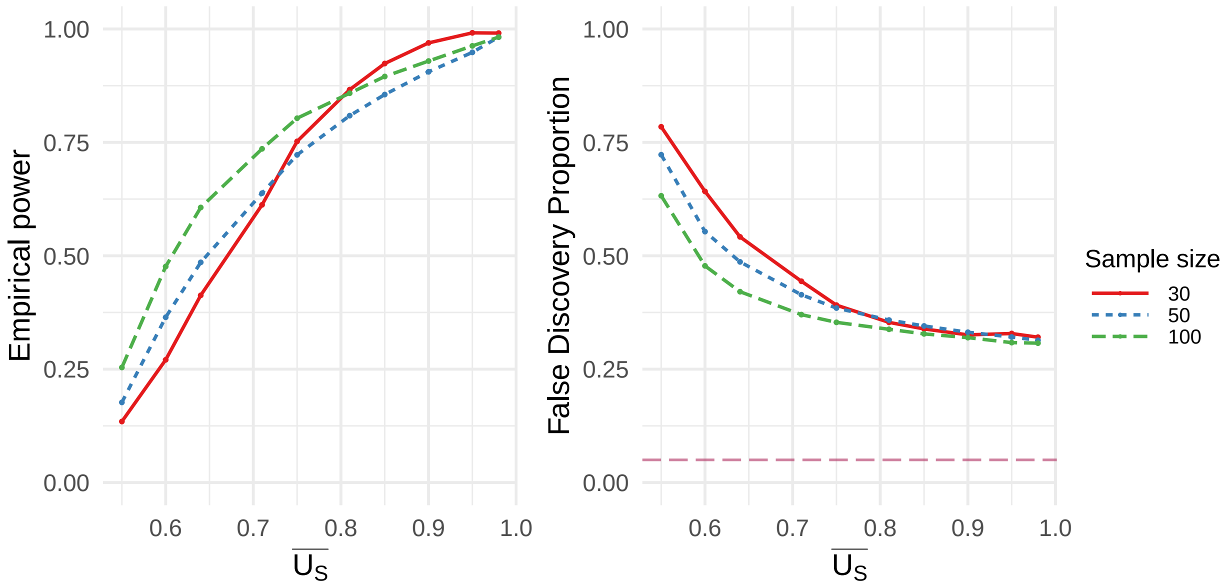

Supporting Information Figure 5: Data generation process 2, scenario 2: empirical power (left) and false discovery proportion (right) prior to multiple testing corrections as a function of average surrogate strength for three different sample sizes. The nominal significance level $\alpha = 0.05$ is plotted as a dashed purple line on the FDR plot.

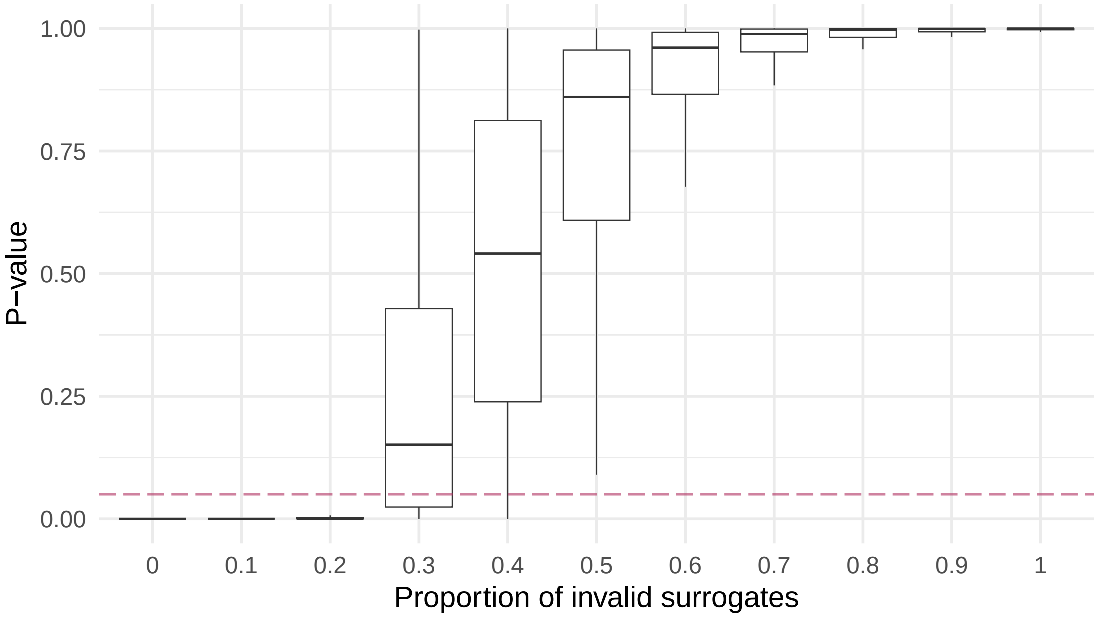

Supporting Information Figure 6: Data generation process 2: The distributions of the p-values in the evaluation step are examined as a function of the false discovery proportion which make up $\widehat{\gamma}_{\mathcal{S}}$, which consists of a combination of 20 predictors. The sample size is $n = 50$ and the valid surrogate strength is $\widehat{U_{S_j}} = 0.9$. The nominal significance level $\alpha = 0.05$ is plotted as a dashed purple line. Desired power for the new surrogate was fixed at 80%.

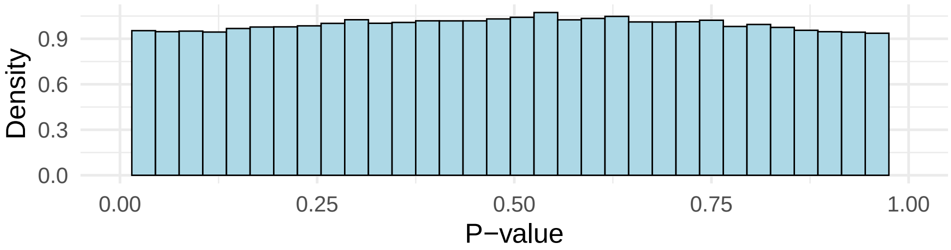

Supporting Information Figure 7: Data generation process 1: distribution of raw p-values under the null hypothesis. The sample size is $n = 50$, the predictors were generated without correlation, and the histogram represents the results across 1000 simulations.

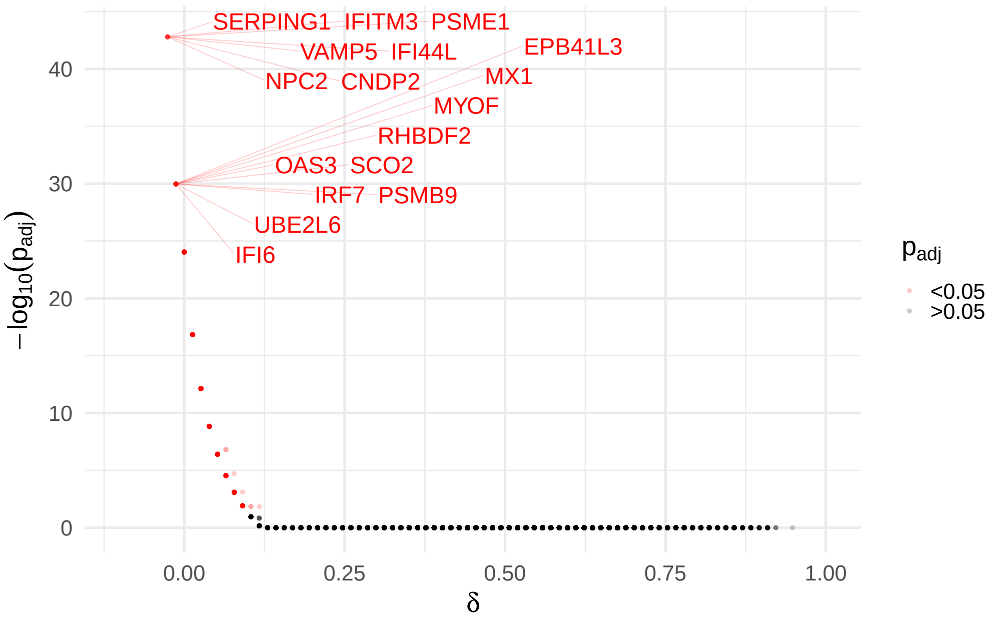

Supporting Information Figure 8: A visual method to select markers to pass the screening stage. The x-axis are the δ values, and the y-axis is the negative log10 of the adjusted p-value. Markers with a stronger surrogate strength appear towards the top-left of the plot. The 222-genes with an adjusted p-value less than 0.05 are highlighted in red- note that many points are on top of each other due to equivalent p-values resulting from the paired sample test.

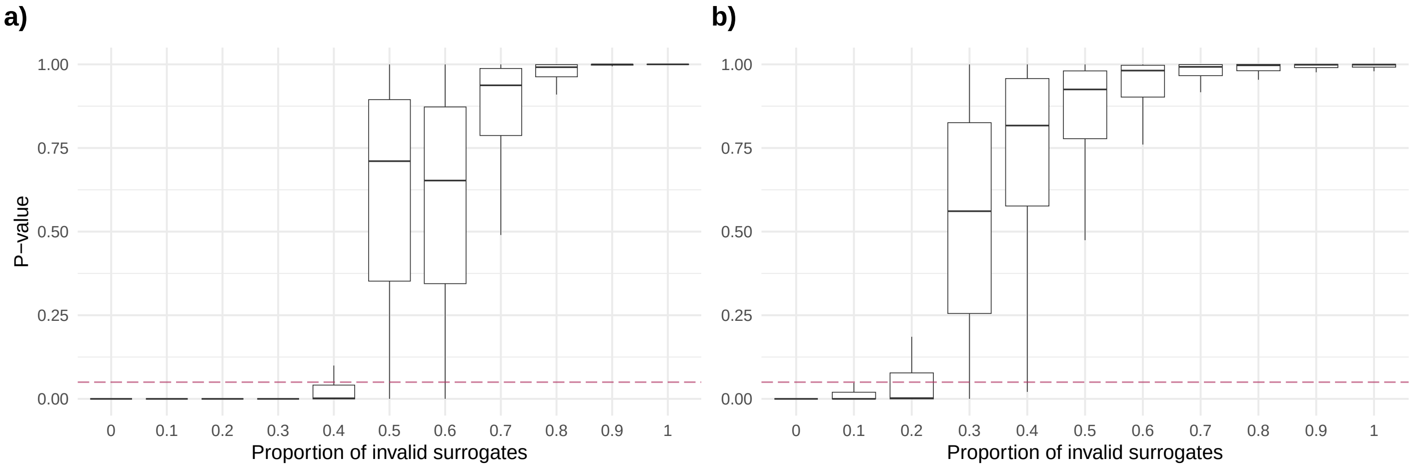

Supporting Information Figure 9: The distributions of the p-values in the evaluation step are examined as a function of the false discovery proportion which make up $\widehat{\gamma}_{\mathcal{S}}$, which consists of a combination of a) 100 predictors and b) 10 predictors. The sample size is $n = 50$ and the valid surrogate strength is $\widehat{U_{S_j}} = 0.9$. The nominal significance level $\alpha = 0.05$ is plotted as a dashed purple line. Desired power for the new surrogate was fixed at 80%.

Supporting Information Table 1: Screening results from the data application - all genes with adjusted p-values less than 0.05.

Gene	δ (95% C.I.)	σ_δ	Unadjusted p-value	Bonferroni Adjusted p-value
CNDP2	-0.026 (-0.056, 0.004)	0.018	1.6e-47	1.6e-43
IFI44L	-0.026 (-0.056, 0.004)	0.018	1.6e-47	1.6e-43
IFITM3	-0.026 (-0.056, 0.004)	0.018	1.6e-47	1.6e-43
NPC2	-0.026 (-0.056, 0.004)	0.018	1.6e-47	1.6e-43
PSME1	-0.026 (-0.056, 0.004)	0.018	1.6e-47	1.6e-43
SERPING1	-0.026 (-0.056, 0.004)	0.018	1.6e-47	1.6e-43
VAMP5	-0.026 (-0.056, 0.004)	0.018	1.6e-47	1.6e-43
EPB41L3	-0.013 (-0.05, 0.024)	0.023	1.1e-34	1.1e-30
IFI6	-0.013 (-0.05, 0.024)	0.023	1.1e-34	1.1e-30
IRF7	-0.013 (-0.05, 0.024)	0.023	1.1e-34	1.1e-30
MX1	-0.013 (-0.05, 0.024)	0.023	1.1e-34	1.1e-30
MYOF	-0.013 (-0.05, 0.024)	0.023	1.1e-34	1.1e-30
OAS3	-0.013 (-0.05, 0.024)	0.023	1.1e-34	1.1e-30
PSMB9	-0.013 (-0.05, 0.024)	0.023	1.1e-34	1.1e-30
RHBDF2	-0.013 (-0.05, 0.024)	0.023	1.1e-34	1.1e-30
SCO2	-0.013 (-0.05, 0.024)	0.023	1.1e-34	1.1e-30
UBE2L6	-0.013 (-0.05, 0.024)	0.023	1.1e-34	1.1e-30
WARS1	-0.013 (-0.05, 0.024)	0.023	1.1e-34	1.1e-30
ADAP2	0 (-0.043, 0.043)	0.026	9.1e-29	9.2e-25
BST2	0 (-0.043, 0.043)	0.026	9.1e-29	9.2e-25
CEACAM1	0 (-0.043, 0.043)	0.026	9.1e-29	9.2e-25
CYBB	0 (-0.043, 0.043)	0.026	9.1e-29	9.2e-25
HERC5	0 (-0.043, 0.043)	0.026	9.1e-29	9.2e-25
IFI35	0 (-0.043, 0.043)	0.026	9.1e-29	9.2e-25
IFIH1	0 (-0.043, 0.043)	0.026	9.1e-29	9.2e-25
IFITM1	0 (-0.043, 0.043)	0.026	9.1e-29	9.2e-25
LY6E	0 (-0.043, 0.043)	0.026	9.1e-29	9.2e-25
MICB	0 (-0.043, 0.043)	0.026	9.1e-29	9.2e-25
NAGK	0 (-0.043, 0.043)	0.026	9.1e-29	9.2e-25
OAS1	0 (-0.043, 0.043)	0.026	9.1e-29	9.2e-25
OASL	0 (-0.043, 0.043)	0.026	9.1e-29	9.2e-25
P2RX7	0 (-0.043, 0.043)	0.026	9.1e-29	9.2e-25
PSMB10	0 (-0.043, 0.043)	0.026	9.1e-29	9.2e-25
RSAD2	0 (-0.043, 0.043)	0.026	9.1e-29	9.2e-25
RTP4	0 (-0.043, 0.043)	0.026	9.1e-29	9.2e-25
SCARB2	0 (-0.043, 0.043)	0.026	9.1e-29	9.2e-25
SQOR	0 (-0.043, 0.043)	0.026	9.1e-29	9.2e-25
STAT2	0 (-0.043, 0.043)	0.026	9.1e-29	9.2e-25
TLR7	0 (-0.043, 0.043)	0.026	9.1e-29	9.2e-25
TRIM21	0 (-0.043, 0.043)	0.026	9.1e-29	9.2e-25
TYMP	0 (-0.043, 0.043)	0.026	9.1e-29	9.2e-25
XAF1	0 (-0.043, 0.043)	0.026	9.1e-29	9.2e-25
ADAR	0.013 (-0.035, 0.061)	0.029	1.5e-21	1.5e-17
AFF1	0.013 (-0.035, 0.061)	0.029	1.5e-21	1.5e-17
ATP1B3	0.013 (-0.035, 0.061)	0.029	1.5e-21	1.5e-17
DHRS9	0.013 (-0.035, 0.061)	0.029	1.5e-21	1.5e-17
EIF2AK2	0.013 (-0.035, 0.061)	0.029	1.5e-21	1.5e-17
GBP1	0.013 (-0.035, 0.061)	0.029	1.5e-21	1.5e-17
GBP2	0.013 (-0.035, 0.061)	0.029	1.5e-21	1.5e-17
GCH1	0.013 (-0.035, 0.061)	0.029	1.5e-21	1.5e-17
GNS	0.013 (-0.035, 0.061)	0.029	1.5e-21	1.5e-17
GSDMD	0.013 (-0.035, 0.061)	0.029	1.5e-21	1.5e-17
IFIT3	0.013 (-0.035, 0.061)	0.029	1.5e-21	1.5e-17
MAFB	0.013 (-0.035, 0.061)	0.029	1.5e-21	1.5e-17
MT2A	0.013 (-0.035, 0.061)	0.029	1.5e-21	1.5e-17
NOD2	0.013 (-0.035, 0.061)	0.029	1.5e-21	1.5e-17
OAS2	0.013 (-0.035, 0.061)	0.029	1.5e-21	1.5e-17
RBCK1	0.013 (-0.035, 0.061)	0.029	1.5e-21	1.5e-17
SHTN1	0.013 (-0.035, 0.061)	0.029	1.5e-21	1.5e-17
SRBD1	0.013 (-0.035, 0.061)	0.029	1.5e-21	1.5e-17
STAT1	0.013 (-0.035, 0.061)	0.029	1.5e-21	1.5e-17
TBC1D2B	0.013 (-0.035, 0.061)	0.029	1.5e-21	1.5e-17
TNF	0.013 (-0.035, 0.061)	0.029	1.5e-21	1.5e-17
TNFAIP6	0.013 (-0.035, 0.061)	0.029	1.5e-21	1.5e-17
AKR1A1	0.026 (-0.026, 0.078)	0.032	7.3e-17	7.3e-13
ALDH1A1	0.026 (-0.026, 0.078)	0.032	7.3e-17	7.3e-13
ALDH2	0.026 (-0.026, 0.078)	0.032	7.3e-17	7.3e-13
ARSB	0.026 (-0.026, 0.078)	0.032	7.3e-17	7.3e-13

**Gene**	δ **(95% C.I.)**	σ_δ	**Unadjusted p-value**	**Bonferroni Adjusted p-value**
ATF5	0.026 (-0.026, 0.078)	0.032	7.3e-17	7.3e-13
CALCOCO2	0.026 (-0.026, 0.078)	0.032	7.3e-17	7.3e-13
DDX58	0.026 (-0.026, 0.078)	0.032	7.3e-17	7.3e-13
DENND1A	0.026 (-0.026, 0.078)	0.032	7.3e-17	7.3e-13
DRAP1	0.026 (-0.026, 0.078)	0.032	7.3e-17	7.3e-13
HLA_F	0.026 (-0.026, 0.078)	0.032	7.3e-17	7.3e-13
IFI44	0.026 (-0.026, 0.078)	0.032	7.3e-17	7.3e-13
IFIT2	0.026 (-0.026, 0.078)	0.032	7.3e-17	7.3e-13
IRF9	0.026 (-0.026, 0.078)	0.032	7.3e-17	7.3e-13
KYNU	0.026 (-0.026, 0.078)	0.032	7.3e-17	7.3e-13
LHFPL2	0.026 (-0.026, 0.078)	0.032	7.3e-17	7.3e-13
MSRB2	0.026 (-0.026, 0.078)	0.032	7.3e-17	7.3e-13
MTMR11	0.026 (-0.026, 0.078)	0.032	7.3e-17	7.3e-13
PLSCR1	0.026 (-0.026, 0.078)	0.032	7.3e-17	7.3e-13
SLC2A6	0.026 (-0.026, 0.078)	0.032	7.3e-17	7.3e-13
SORT1	0.026 (-0.026, 0.078)	0.032	7.3e-17	7.3e-13
SP110	0.026 (-0.026, 0.078)	0.032	7.3e-17	7.3e-13
SP140	0.026 (-0.026, 0.078)	0.032	7.3e-17	7.3e-13
STX11	0.026 (-0.026, 0.078)	0.032	7.3e-17	7.3e-13
TDRD7	0.026 (-0.026, 0.078)	0.032	7.3e-17	7.3e-13
TENT5A	0.026 (-0.026, 0.078)	0.032	7.3e-17	7.3e-13
TRAFD1	0.026 (-0.026, 0.078)	0.032	7.3e-17	7.3e-13
UNC93B1	0.026 (-0.026, 0.078)	0.032	7.3e-17	7.3e-13
ASGR2	0.039 (-0.017, 0.095)	0.034	1.4e-13	1.5e-09
ATOX1	0.039 (-0.017, 0.095)	0.034	1.4e-13	1.5e-09
C1QB	0.039 (-0.017, 0.095)	0.034	1.4e-13	1.5e-09
CD300A	0.039 (-0.017, 0.095)	0.034	1.4e-13	1.5e-09
DRAM1	0.039 (-0.017, 0.095)	0.034	1.4e-13	1.5e-09
DUSP3	0.039 (-0.017, 0.095)	0.034	1.4e-13	1.5e-09
DUSP5	0.039 (-0.017, 0.095)	0.034	1.4e-13	1.5e-09
EMILIN2	0.039 (-0.017, 0.095)	0.034	1.4e-13	1.5e-09
IRF1	0.039 (-0.017, 0.095)	0.034	1.4e-13	1.5e-09
ISG20	0.039 (-0.017, 0.095)	0.034	1.4e-13	1.5e-09
MX2	0.039 (-0.017, 0.095)	0.034	1.4e-13	1.5e-09
P2RY14	0.039 (-0.017, 0.095)	0.034	1.4e-13	1.5e-09
PANK2	0.039 (-0.017, 0.095)	0.034	1.4e-13	1.5e-09
PARP12	0.039 (-0.017, 0.095)	0.034	1.4e-13	1.5e-09
PLEK	0.039 (-0.017, 0.095)	0.034	1.4e-13	1.5e-09
PLEKHO1	0.039 (-0.017, 0.095)	0.034	1.4e-13	1.5e-09
PSMB2	0.039 (-0.017, 0.095)	0.034	1.4e-13	1.5e-09
PSTPIP2	0.039 (-0.017, 0.095)	0.034	1.4e-13	1.5e-09
SAMD4A	0.039 (-0.017, 0.095)	0.034	1.4e-13	1.5e-09
SLC6A12	0.039 (-0.017, 0.095)	0.034	1.4e-13	1.5e-09
SNTB1	0.039 (-0.017, 0.095)	0.034	1.4e-13	1.5e-09
SPATS2L	0.039 (-0.017, 0.095)	0.034	1.4e-13	1.5e-09
TFIP11	0.039 (-0.017, 0.095)	0.034	1.4e-13	1.5e-09
TIMM10	0.039 (-0.017, 0.095)	0.034	1.4e-13	1.5e-09
TNFAIP2	0.039 (-0.017, 0.095)	0.034	1.4e-13	1.5e-09
TRIM5	0.039 (-0.017, 0.095)	0.034	1.4e-13	1.5e-09
FGR	0.065 (0.009, 0.12)	0.034	1.5e-11	1.5e-07
HERC6	0.065 (0.009, 0.12)	0.034	1.5e-11	1.5e-07
AIM2	0.052 (-0.008, 0.112)	0.036	3.9e-11	3.9e-07
ANKFY1	0.052 (-0.008, 0.112)	0.036	3.9e-11	3.9e-07
ATF3	0.052 (-0.008, 0.112)	0.036	3.9e-11	3.9e-07
BLVRA	0.052 (-0.008, 0.112)	0.036	3.9e-11	3.9e-07
CTNNA1	0.052 (-0.008, 0.112)	0.036	3.9e-11	3.9e-07
CXCL10	0.052 (-0.008, 0.112)	0.036	3.9e-11	3.9e-07
DDX60	0.052 (-0.008, 0.112)	0.036	3.9e-11	3.9e-07
DHX58	0.052 (-0.008, 0.112)	0.036	3.9e-11	3.9e-07
DPYD	0.052 (-0.008, 0.112)	0.036	3.9e-11	3.9e-07
FAM111A	0.052 (-0.008, 0.112)	0.036	3.9e-11	3.9e-07
HLA_DMA	0.052 (-0.008, 0.112)	0.036	3.9e-11	3.9e-07
IFIT1	0.052 (-0.008, 0.112)	0.036	3.9e-11	3.9e-07
IRF2	0.052 (-0.008, 0.112)	0.036	3.9e-11	3.9e-07
KCNJ2	0.052 (-0.008, 0.112)	0.036	3.9e-11	3.9e-07
LILRB2	0.052 (-0.008, 0.112)	0.036	3.9e-11	3.9e-07
NFKBIE	0.052 (-0.008, 0.112)	0.036	3.9e-11	3.9e-07
PHF11	0.052 (-0.008, 0.112)	0.036	3.9e-11	3.9e-07
PLAGL1	0.052 (-0.008, 0.112)	0.036	3.9e-11	3.9e-07
PSMB8	0.052 (-0.008, 0.112)	0.036	3.9e-11	3.9e-07
SRC	0.052 (-0.008, 0.112)	0.036	3.9e-11	3.9e-07

Gene	δ (95% C.I.)	σ_δ	Unadjusted p-value	Bonferroni Adjusted p-value
TAPBPL	0.052 (-0.008, 0.112)	0.036	3.9e-11	3.9e-07
TRIM22	0.052 (-0.008, 0.112)	0.036	3.9e-11	3.9e-07
DECR1	0.078 (0.019, 0.137)	0.036	1.9e-09	1.9e-05
ACTA2	0.065 (0.002, 0.128)	0.039	2.8e-09	2.8e-05
CD300C	0.065 (0.002, 0.128)	0.039	2.8e-09	2.8e-05
CTRL	0.065 (0.002, 0.128)	0.039	2.8e-09	2.8e-05
CTSS	0.065 (0.002, 0.128)	0.039	2.8e-09	2.8e-05
FCN1	0.065 (0.002, 0.128)	0.039	2.8e-09	2.8e-05
HEBP1	0.065 (0.002, 0.128)	0.039	2.8e-09	2.8e-05
HLA_DMB	0.065 (0.002, 0.128)	0.039	2.8e-09	2.8e-05
HLA_DPA1	0.065 (0.002, 0.128)	0.039	2.8e-09	2.8e-05
HLA_DRA	0.065 (0.002, 0.128)	0.039	2.8e-09	2.8e-05
IFI16	0.065 (0.002, 0.128)	0.039	2.8e-09	2.8e-05
IL15	0.065 (0.002, 0.128)	0.039	2.8e-09	2.8e-05
KCNMB1	0.065 (0.002, 0.128)	0.039	2.8e-09	2.8e-05
KLF4	0.065 (0.002, 0.128)	0.039	2.8e-09	2.8e-05
MICU1	0.065 (0.002, 0.128)	0.039	2.8e-09	2.8e-05
NUCB1	0.065 (0.002, 0.128)	0.039	2.8e-09	2.8e-05
OGFR	0.065 (0.002, 0.128)	0.039	2.8e-09	2.8e-05
PLAAT4	0.065 (0.002, 0.128)	0.039	2.8e-09	2.8e-05
PLAGL2	0.065 (0.002, 0.128)	0.039	2.8e-09	2.8e-05
PSMA5	0.065 (0.002, 0.128)	0.039	2.8e-09	2.8e-05
REC8	0.065 (0.002, 0.128)	0.039	2.8e-09	2.8e-05
TCN2	0.065 (0.002, 0.128)	0.039	2.8e-09	2.8e-05
TMEM140	0.065 (0.002, 0.128)	0.039	2.8e-09	2.8e-05
TNS3	0.065 (0.002, 0.128)	0.039	2.8e-09	2.8e-05
DPYSL2	0.091 (0.029, 0.153)	0.038	7.7e-08	7.7e-04
APOBEC3G	0.078 (0.012, 0.144)	0.040	8.1e-08	8.2e-04
ASCL2	0.078 (0.012, 0.144)	0.040	8.1e-08	8.2e-04
ASGR1	0.078 (0.012, 0.144)	0.040	8.1e-08	8.2e-04
BTN3A3	0.078 (0.012, 0.144)	0.040	8.1e-08	8.2e-04
CD74	0.078 (0.012, 0.144)	0.040	8.1e-08	8.2e-04
CDC42EP2	0.078 (0.012, 0.144)	0.040	8.1e-08	8.2e-04
CTSL	0.078 (0.012, 0.144)	0.040	8.1e-08	8.2e-04
CUL1	0.078 (0.012, 0.144)	0.040	8.1e-08	8.2e-04
DMXL2	0.078 (0.012, 0.144)	0.040	8.1e-08	8.2e-04
ETV6	0.078 (0.012, 0.144)	0.040	8.1e-08	8.2e-04
FAR2	0.078 (0.012, 0.144)	0.040	8.1e-08	8.2e-04
FFAR2	0.078 (0.012, 0.144)	0.040	8.1e-08	8.2e-04
FYB1	0.078 (0.012, 0.144)	0.040	8.1e-08	8.2e-04
GADD45B	0.078 (0.012, 0.144)	0.040	8.1e-08	8.2e-04
GAS6	0.078 (0.012, 0.144)	0.040	8.1e-08	8.2e-04
GSTK1	0.078 (0.012, 0.144)	0.040	8.1e-08	8.2e-04
IL12RB1	0.078 (0.012, 0.144)	0.040	8.1e-08	8.2e-04
ILK	0.078 (0.012, 0.144)	0.040	8.1e-08	8.2e-04
IRF5	0.078 (0.012, 0.144)	0.040	8.1e-08	8.2e-04
KPNB1	0.078 (0.012, 0.144)	0.040	8.1e-08	8.2e-04
LILRB4	0.078 (0.012, 0.144)	0.040	8.1e-08	8.2e-04
MRPL44	0.078 (0.012, 0.144)	0.040	8.1e-08	8.2e-04
MYD88	0.078 (0.012, 0.144)	0.040	8.1e-08	8.2e-04
PARP3	0.078 (0.012, 0.144)	0.040	8.1e-08	8.2e-04
PML	0.078 (0.012, 0.144)	0.040	8.1e-08	8.2e-04
RIPK2	0.078 (0.012, 0.144)	0.040	8.1e-08	8.2e-04
RNF114	0.078 (0.012, 0.144)	0.040	8.1e-08	8.2e-04
RRAS	0.078 (0.012, 0.144)	0.040	8.1e-08	8.2e-04
SCPEP1	0.078 (0.012, 0.144)	0.040	8.1e-08	8.2e-04
SEC24D	0.078 (0.012, 0.144)	0.040	8.1e-08	8.2e-04
SLC20A1	0.078 (0.012, 0.144)	0.040	8.1e-08	8.2e-04
SLC27A3	0.078 (0.012, 0.144)	0.040	8.1e-08	8.2e-04
SLC7A7	0.078 (0.012, 0.144)	0.040	8.1e-08	8.2e-04
TAPBP	0.078 (0.012, 0.144)	0.040	8.1e-08	8.2e-04
TOR1B	0.078 (0.012, 0.144)	0.040	8.1e-08	8.2e-04
ACOT9	0.091 (0.022, 0.16)	0.042	1.2e-06	1.2e-02
ACSL5	0.091 (0.022, 0.16)	0.042	1.2e-06	1.2e-02
APOL6	0.091 (0.022, 0.16)	0.042	1.2e-06	1.2e-02
BID	0.091 (0.022, 0.16)	0.042	1.2e-06	1.2e-02
CASP5	0.091 (0.022, 0.16)	0.042	1.2e-06	1.2e-02
CASZ1	0.091 (0.022, 0.16)	0.042	1.2e-06	1.2e-02
CD40	0.091 (0.022, 0.16)	0.042	1.2e-06	1.2e-02
ETV7	0.091 (0.022, 0.16)	0.042	1.2e-06	1.2e-02
IL18BP	0.091 (0.022, 0.16)	0.042	1.2e-06	1.2e-02

Gene	δ (95% C.I.)	σ_δ	Unadjusted p-value	Bonferroni Adjusted p-value
KCNJ15	0.091 (0.022, 0.16)	0.042	1.2e-06	1.2e-02
LAMP3	0.091 (0.022, 0.16)	0.042	1.2e-06	1.2e-02
LGALS3BP	0.091 (0.022, 0.16)	0.042	1.2e-06	1.2e-02
LMO2	0.091 (0.022, 0.16)	0.042	1.2e-06	1.2e-02
LTBR	0.091 (0.022, 0.16)	0.042	1.2e-06	1.2e-02
PDK3	0.091 (0.022, 0.16)	0.042	1.2e-06	1.2e-02
PSMB3	0.091 (0.022, 0.16)	0.042	1.2e-06	1.2e-02
RTN1	0.091 (0.022, 0.16)	0.042	1.2e-06	1.2e-02
STAT5A	0.091 (0.022, 0.16)	0.042	1.2e-06	1.2e-02
MARCO	0.104 (0.039, 0.169)	0.040	1.4e-06	1.4e-02
ZFYVE26	0.104 (0.039, 0.169)	0.040	1.4e-06	1.4e-02
BTN3A1	0.117 (0.056, 0.178)	0.037	1.4e-06	1.5e-02

Supporting Information Table S2: Sensitivity analysis evaluating the effect of varying the non-inferiority margin ϵ, where values closer to 0 result in fewer candidate surrogates to combine for the evaluation stage. The evaluation metric for the combined marker, $\boldsymbol{\delta_{\gamma_S}}$, its standard deviation, and its p-value corresponding to a test based on a desired power of 90% are given in the table.

ϵ (screening)	No. of genes in γ_S	δ_{γ_S} (95% C.I.)	$\sigma_{\delta_{\gamma_S}}$	p-value
0.05	0			
0.1	0			
0.15	64	-0.038 (-0.102, 0.025)	0.038	3.1e-03
0.20	117	-0.038 (-0.102, 0.025)	0.038	3.1e-03
0.25	165	-0.038 (-0.102, 0.025)	0.038	3.1e-03
0.30	246	-0.038 (-0.102, 0.025)	0.038	3.1e-03
0.35	301	-0.038 (-0.102, 0.025)	0.038	3.1e-03

Supporting Information Table S3: Comparison of evaluation results between the composite surrogate marker γ_S, constructed from the 222 significant genes identified in the screening stage, and the top 10 genes from the screening stage evaluated individually in the evaluation data.

Marker	δ	σ	p-value
γ_S	-0.038 (-0.102, 0.025)	0.038	3.1e-03
CNDP2	0 (-0.091, 0.091)	0.055	4.8e-03
IFI44L	-0.038 (-0.102, 0.025)	0.038	3.1e-03
IFITM3	-0.038 (-0.102, 0.025)	0.038	3.1e-03
NPC2	-0.038 (-0.102, 0.025)	0.038	3.1e-03
PSME1	-0.038 (-0.102, 0.025)	0.038	3.1e-03
SERPING1	-0.038 (-0.102, 0.025)	0.038	3.1e-03
VAMP5	-0.038 (-0.102, 0.025)	0.038	3.1e-03
EPB41L3	-0.038 (-0.102, 0.025)	0.038	3.1e-03
IFI6	-0.038 (-0.102, 0.025)	0.038	3.1e-03
IRF7	-0.038 (-0.102, 0.025)	0.038	3.1e-03
